# Investigation of mechanical properties, remineralization, antibacterial effect, and cellular toxicity of composite orthodontic adhesive combined with silver-containing nanostructured bioactive glass

**DOI:** 10.1186/s12903-024-04402-7

**Published:** 2024-06-01

**Authors:** Massoud Seifi, Fatemeh Eskandarloo, Parisa Amdjadi, Abbas Farmany

**Affiliations:** 1 Dentofacial Deformities Research Center, Research Institute of Dental Sciences, School of Dentistry, Shahid Beheshti University of Medical Sciences, Tehran, Iran; 2 Department of Orthodontics, School of Dentistry, Hamadan University of Medical Sciences, Hamadan, Iran; 3 Department of Dental Biomaterials, School of Dentistry, Shahid Beheshti University of Medical Sciences, Tehran, Iran; 4 Dental Implant Research Center, School of Dentistry, Hamadan University of Medical Sciences, Hamadan, Iran

**Keywords:** Remineralization, Orthodontic adhesive, Shear bond strength, Bioactive glass, Nanoparticles

## Abstract

**Background:**

The formation of white spots, which represent early carious lesions, is a major issue with fixed orthodontics. The addition of remineralizing agents to orthodontic adhesives may prevent the formation of white spots. The aim of this study was to produce a composite orthodontic adhesive combined with nano-bioactive glass-silver (nBG@Ag) for bracket bonding to enamel and to investigate its cytotoxicity, antimicrobial activity, remineralization capability, and bond strength.

**Methods:**

nBG@Ag was synthesized using the sol-gel method, and characterized using transmission electron microscopy (TEM), X-ray diffraction (XRD), and Fourier-transform infrared spectroscopy with an attenuated total reflectance attachment (ATR-FTIR). The cytotoxicity test (MTT) and antimicrobial activity of adhesives containing 1%, 3%, and 5% (*wt/wt*) nBG@Ag were evaluated, and the shear bond strength of the adhesives was measured using a universal testing machine. Remineralization was assessed through microhardness testing with a Vickers microhardness tester and scanning electron microscopy (SEM). Statistical analyses were conducted using the Shapiro-Wilk test, Levene test, one-way ANOVA, Robust-Welch test, Tukey HSD method, and two-way ANOVA.

**Results:**

The biocompatibility of the adhesives was found to be high, as confirmed by the lack of significant differences in the cytotoxicity between the sample and control groups. Discs made from composites containing nBG@Ag exhibited a significant reduction in the growth of *Streptococcus mutans* (*p* < 0.05), and the antibacterial activity increased with higher percentages of nBG@Ag. The shear bond strength of the adhesives decreased significantly (*p* < 0.001) after the addition of nanoparticles, but it remained above the recommended value. The addition of nBG@Ag showed improvement in the microhardness of the teeth, although the differences in microhardness between the study groups were not statistically significant. The formation of hydroxyapatite deposits on the tooth surface was confirmed through SEM and energy-dispersive X-ray spectroscopy (EDX).

**Conclusion:**

Adding nBG@Ag to orthodontic adhesives can be an effective approach to enhance antimicrobial activity and reduce enamel demineralization around the orthodontic brackets, without compromising biocompatibility and bond strength.

## Introduction

During orthodontic treatment, the occurrence of enamel demineralization or white spot lesions is a common issue, affecting a significant percentage of orthodontic patients [1–3]. This is primarily due to poor oral hygiene compliance and the increased surfaces available for bacterial biofilm formation caused by fixed orthodontic appliances [1–4]. Previous studies have suggested various methods to prevent or reduce the formation of white spot lesions, including oral hygiene instruction, selective acid etching technique, and fluoride application [5, 6]. Additionally, localized remineralizing agents have been used to restore dental enamel [7]. Fluoride, for example, can inhibit demineralization by forming fluorapatite on the enamel surface, thus increasing its resistance to acid attacks [8]. Different forms of fluoride delivery, such as varnishes, toothpaste, mouth rinses, solutions, gels, and orthodontic adhesives containing fluoride, have been employed, although most of these methods require patient cooperation [9]. In recent years, casein phosphopeptide-amorphous calcium phosphate (CPP-ACP) has gained popularity as an alternative to fluoride for enamel remineralization [7]. By adhering to dental biofilm, CPP-ACP prevents bacterial colonization and creates a supersaturated environment of calcium and phosphate ions [10, 11]. However, clinical studies have shown limited effectiveness of CPP-ACP, with only slight differences compared to fluoride use [12, 13]. Bioactive glass (45S5), a material developed for dental applications, has shown promise in remineralizing white spot lesions around brackets [14–20]. When in contact with saliva or other physiological fluids, bioactive glass induces the formation of apatite on the enamel surface [21]. Its antibacterial activity is another significant property, particularly in the case of silver-containing bioactive glass, which has demonstrated remarkable anti-caries effects [21]. In an animal study, the optimal concentration of silver particles for antimicrobial properties was found to be 5% [22]. Previous studies primarily focused on adding antibacterial and remineralizing agents to orthodontic primers. However, in this study, a combined nanoparticle was incorporated directly into the orthodontic adhesive, reducing the clinical steps during bracket bonding. Since a composite orthodontic adhesive with satisfactory bond strength and biocompatibility for effective prevention of white spot lesions has yet to be introduced.

The objective of this study was to develop a composite orthodontic adhesive combined with nanostructured bioactive glass containing silver for bracket bonding to dental enamel. The study aimed to evaluate its cytotoxicity, remineralization potential, mechanical properties, and antibacterial activity, comparing it with conventional adhesive.

## Materials and methods

This in vitro study received ethical approval from the committee of Shahid Beheshti University of Medical Sciences with the ID IR.SBMU.DRC.REC.1402.081.

The materials utilized in this study included the orthodontic composite (Adhesive) (GC Ortho Connect, GC Orthodontics, Japan). Additionally, an experimental-synthesized composite was prepared, consisting of equal proportions of Bis-GMA and TEGDMA (Merck KgaA, Darmstadt, Germany), along with 75% *w/w* silanized silica. To create different formulations, 1%, 3%, and 5% *w/w* nBG@Ag were added to both types of composites. The samples were classified into eight groups, as follows:

1. Experimental orthodontic composite (E) (control).
2. Experimental orthodontic composite containing 1% nBG@Ag.
3. Experimental orthodontic composite containing 3% nBG@Ag.
4. Experimental orthodontic composite containing 5% nBG@Ag.
5. GC Ortho Connect orthodontic composite (control).
6. GC orthodontic composite containing 1% nBG@Ag.
7. GC orthodontic composite containing 3% nBG@Ag.
8. GC orthodontic composite containing 5% nBG@Ag.

In this study, extracted healthy human premolar teeth and discs made from adhesives were used to investigate the cytotoxicity, remineralization ability, mechanical properties, and antibacterial properties of synthesized adhesives.

To determine the appropriate sample size, certain parameters were considered. The study aimed for a type I error (α) of 0.05 and a type II error (β) of 0.2, which corresponds to a test power of 80%. Mean and standard deviation values of the variable under investigation were obtained from the literature [18]. From these values, the effect size was calculated as 0.464. Based on these considerations, the sample size in each group was determined to be 10 teeth, resulting in a total of 80 teeth. To account for potential sample loss during the study, 12 teeth were included in each group, leading to a total of 80 teeth. However, for statistical calculations, only 10 teeth from each group were considered. For the cytotoxicity and antimicrobial tests, 5 adhesive discs were selected in each group.

### Synthesis of nano-bioglass-silver

The synthesis of nano-bioglass-silver (nBG@Ag) was conducted using the sol-gel method [11, 23]. Initially, a mixture of tetraethyl orthosilicate (TEOS) and calcium nitrate in a water/ethanol solution (at a ratio of 2:1) was prepared. To adjust the pH of the solution to 2, 1 M citric acid was added. The resulting solution, referred to as A, was mixed until it became clear and homogeneous. In parallel, a 2% solution of polyethylene glycol (PEG) with a molecular weight of 2000 and di-ammonium hydrogen orthophosphate were prepared. Specific amounts of silver nitrate ion was incorporated into the solution. By adding ammonia, the pH of the solution, known as B, was adjusted to 10.

Next, A and B were combined and mixed for 10 h to ensure the formation of a homogeneous gel. Afterward, the gel was washed with deionized water to remove impurities. Subsequently, the gel underwent drying and lyophilization to eliminate the solvent. Finally, annealing was carried out at a temperature of 650 °C for 10 h to facilitate crystallization and achieve the desired nano-bioglass structure. For the incorporation of nBG@Ag into the orthodontic composite, different percentages (1%, 3%, and 5% *w/w*) of the nanoparticles were added to composites: the commercially available orthodontic composite (adhesive), and an experimental-synthesized composite comprising equal proportions of Bis-GMA and TEGDMA, along with 75% *w/w* silanized silica. The addition of the nanoparticle percentages was accomplished by mixing the composites for one minute using a spatula and glass slab [24]. The mixing process was continued until a fully homogeneous mixture was obtained.

### Characterization of nanoparticles

To examine the crystalline structure of nBG@Ag, X-ray diffraction (XRD) analysis was performed. For this purpose, a PANalytical Xpert PRO X-ray diffractometer (Netherlands- Xpert Pro MPD) was used. The X-ray tube used was Cu Kα radiation with a wavelength of 1.54 angstroms and a nickel filter. The applied voltage was 40 kilovolts and the equivalent current was 35 milliamperes.

To evaluate the functional groups, chemical structure, and chemical bonding states of the synthesized nBG@Ag, Fourier-transform infrared spectroscopy using an attenuated total reflectance attachment (ATR-FTIR) was utilized. The ATR-FTIR spectrum of the synthesized nBG@Ag was recorded in the range of 400–4000 cm^-1^.

In order to examine the shape and size of the nanoparticles, transmission electron microscopy (TEM) was employed using a Carl Zeiss EM900 microscope (Oberkochen, Germany).

To assess the cytotoxicity and antimicrobial activity, adhesive discs with a diameter of 6 mm and a depth of 2 mm was prepared and light cured.

### Cytotoxicity assay

To evaluate the cytotoxicity of the compounds, the Vero cell line, which exhibits a fibroblast-like structure, was employed. For the assay, activated methylthiazole tetrazolium (MTT) solution was prepared by dissolving 5 mg of MTT powder in a tube containing 1000 µl of phosphate-buffered saline (PBS) solution. After thorough mixing and filtration through a 0.22 μm filter, the solution was stored at -20 ℃. In the MTT assay (48 h), 10 µl of the activated MTT solution were added to the Vero cells in each well of the culture plate. The plate was then incubated at 37 ℃ for 4 h. Following the incubation, 100 µl of the culture medium were removed from each well, and 100 µl of dimethyl sulfoxide (DMSO) solution was added. To dissolve the formazan crystals completely, the plate was placed on a Rotator (Pars Azma, Tehran, Iran) for 20 min. After the dissolution step, the optical absorbance of the formazan solution was measured at 570 nm using an ELLISA Plate Reader (Sunrise, Tecan Switzerland). By comparing the toxicity and cell viability levels of the experimental groups (each consisting of 5 adhesive disc samples) with the control sample, the percentage of viable cells was determined using the formula:

$${\rm{Viability}}\left( {\rm{\% }} \right)\,{\rm{ = }}\,{{{\rm{OD}}\,{\rm{Sample}}} \over {{\rm{OD}}\,{\rm{Control}}}}\,{\rm{ \times }}\,{\rm{100}}$$

The calculated biocompatibility percentage was classified according to the Dahl index as follows [25]:

- Cell viability less than 30% = Severe cytotoxicity.
- Cell viability between 30% and 60% = Moderate cytotoxicity.
- Cell viability between 60% and 90% = Slight cytotoxicity.
- Cell viability over 90% = No cytotoxicity.

### Measurement of antimicrobial activity using the microbroth dilution method

To assess the antibacterial activity of bioglass (BG) and nano-bioglass with silver (nBG@Ag) against *S. mutans* (PTC 1643), the minimum inhibitory concentration (MIC) and minimum bactericidal concentration (MBC) were determined following the guidelines provided by the Clinical and Laboratory Standards Institute (CLSI) [26].

The MIC, which indicates the lowest concentration of nBG@Ag (µg mL^-1^) that inhibits the growth of *S. mutans*, was determined using the microbroth dilution method. Sterile 96-well plates were prepared for this purpose. The first well contained 200 µl of nBG@Ag solution (µg mL^-1^), and then 100 µl of Mueller-Hinton broth along with 100 µl from the previous well were added to wells 2–12. Through serial dilution, different concentrations were achieved in each well up to the eleventh well, while the twelfth well served as the control group, containing 100 µl of the growth medium. A bacterial suspension with a concentration of 1.5 × 10^6^ CFU/mL was added to all the wells. The plates were incubated at 35 ℃ for 24 h. After the incubation period, the contents of the wells were inoculated onto agar plates. The MIC was determined as the lowest concentration of nBG@Ag (µg mL^-1^) that visibly inhibited the growth of the microorganisms. The MBC was defined as the lowest concentration of nBG@Ag (µg mL^-1^) necessary to kill the tested bacteria. The well in which bacterial growth was first observed was considered the MIC (minimum inhibitory concentration), while the concentration of the previous well was regarded as the MBC (minimum bactericidal concentration).

Measurement of antibacterial activity using the count method.

In the sterile 96-well plates, 100 µl of Mueller-Hinton broth were added to each well. Subsequently, adhesive discs with a diameter of 6 mm and a depth of 2 mm (5 discs per group) were placed into the wells containing the growth medium. Then, 100 µl of bacterial suspension with a concentration of 1.5 × 10^6^ CFU/mL were added to each well, ensuring the discs were fully submerged. The control group consisted of wells containing only bacteria and the growth medium, without any adhesive discs. The plates were incubated at a temperature of 35 ℃ for 24 h, providing an optimal environment for bacterial growth. After the incubation period, 10 µl of the contents from each well were cultured onto Mueller-Hinton agar plates. These agar plates were then incubated for an additional 24 h at 35 ℃ [26]. Following the incubation on agar plates, the colony-forming units per milliliter (CFU/mL) were determined by counting the visible bacterial colonies.

### Sample preparation

First, 96 healthy upper or lower premolar teeth were collected. They were then immersed in a 0.2% thymol solution for 24 h to ensure disinfection. All the teeth were horizontally mounted using self-polymerizing acrylic material and plastic molds. The mounting was done in such a way that the buccal surface of the teeth (the outer surface facing the cheek or lip) was parallel to the applied force during the shear bond test. The collected teeth were stored in deionized water at a temperature of 37 ℃ for a period of 24 h. To prepare the buccal surface of the teeth for microhardness assessment, a sequential polishing process was conducted. Silicon carbide abrasive paper with different particle sizes (400, 600, 800, 1000, and 1200) was used for this purpose, following a specific order [27]. The abrasive papers were used to gradually remove any surface irregularities and create a smooth and standardized enamel surface. After the initial polishing steps, a final polishing was performed using a slow-speed handpiece and a fine composite polishing material. The microhardness of the buccal enamel surface was measured using the Vickers test. During the measurement, a 2 mm margin around the bracket placement area was defined. To protect the remaining tooth surfaces, they were covered with nail polish, preventing any interference during the microhardness assessment. To maintain the integrity of the study and ensure unbiased analysis, all samples were coded and then randomly divided into eight groups using random number generation in Excel. Afterward, the buccal surface of the teeth was etched with 37% phosphoric acid for a duration of 30 s. Subsequently, the teeth were rinsed with water for a time period equivalent to twice the duration of the acid etching. The rinsing was performed at a distance of 15 –11 cm from the teeth to ensure consistent application across all samples. Finally, the teeth were dried thoroughly using oil-free and moisture-free air. This drying step aimed to eliminate any residual moisture from the tooth surface, preparing it for subsequent procedures or evaluations.

In this study, the evaluation of remineralization and shear bond strength involved examining bonded samples organized into groups of eight. The process of bonding the samples included placing composite material onto the base of an orthodontic bracket (Ortho Organizer, Henry Schein, United States). The bracket was then positioned in the desired location with the appropriate pressure. After the bracket placement, a probe was used to fully adapt the bracket onto the tooth surface by applying pressure in the slot area of the bracket. Any excess composite material in each area was carefully removed using the probe, ensuring a clean and precise application. Subsequently, all the samples were cured for 10 s from four different aspects: mesial, distal, gingival, and occlusal. A light-curing device (Wood-pecker LED table blue, China) with a light intensity of 1000 was used for the curing process.

### Shear bond strength (SBS)

The force required to break the bond was measured using a universal testing machine (Santam STM-20, Tehran, Iran) with a 50 kg load cell at a crosshead speed of 0.5 mm/min [28]. The shear bond strength was then calculated using the following formula:

$$\eqalign{& {\rm{Shear}}\,{\rm{bond}}\,{\rm{strength}} = \cr & {\rm{Bonding}}\,{\rm{force}}\left( {{\rm{Newtons}}} \right)/{\rm{Bracket}}\,{\rm{surface}}\,{\rm{area}}\left( {{\rm{10}}{\rm{.5m}}{{\rm{m}}^{\rm{2}}}} \right) \cr}$$

### Evaluation of remineralization using surface microhardness and scanning electron microscope

Surface microhardness (SMH) was measured using a Vickers surface hardness tester (Zcwik-Roell, Germany). Measurements were taken by applying a 300-g load for 15 s at three different locations with a 500-micron distance [29]. The average of the three values was calculated, and a single value was reported as the Vickers microhardness number (VHN) for each sample [30]. SMH was measured at baseline and after the pH cycling process.

For the pH cycling process, after bracket bonding, the samples were stored in deionized water at 37 ℃ for 24 h. The samples were then immersed in a demineralizing solution with a formula of 2.2 mM CaCl_2_, 2.2 mM NaH_2_PO_4_, and 0.05 M acetic acid at pH 4.4 for 6 h, followed by a remineralizing solution with a formula of 1.5 mM CaCl_2_, 0.9 mM NaH_2_PO_4_, and 0.15 M KCl at pH 7 for 18 h, and this cycle was repeated for two weeks. Between each solution change, the samples were transferred to deionized water, rinsed, and dried. The solutions were changed every 7 days [14, 31].

From each of the 8 groups, one sample was selected for the evaluation of the mineral content. The samples were examined using a Tescan mira 3 scanning electron microscope (SEM) equipped with an EDX analyzer. The surface morphology and mineral content of the samples were evaluated at a 2 mm gingival margin of the brackets and cross-sectional views. The samples were coated with a layer of gold and then examined using the SEM at magnifications of ×1000 and ×5000 with an accelerating voltage of 20 kV.

### Statistical analysis

Statistical analysis was performed using SPSS (Ver. 25) software, and graphs were created using Excel 2016. The Shapiro-Wilk test was used to assess the normality of the data distribution. Additionally, the Levene test was used to examine the equality of variances. For comparing the mean values of initial microhardness, secondary microhardness, microhardness changes, and shear bond strength (SBS) among the 8 groups under investigation, either one-way ANOVA or the Robust-Welch test was used, depending on the presence or absence of variance equality. Pairwise comparisons were conducted using the Tukey HSD method. To examine the effect of two factors, composite and bio-glass-silver, on the dependent variables of microhardness and SBS, a two-way ANOVA was employed. A type I error rate of α = 0.05 was considered for the tests. Therefore, probability values less than this threshold were considered statistically significant.

## Results

### Characterization of bioactive glass-silver nanoparticles

**Fig. 1:**
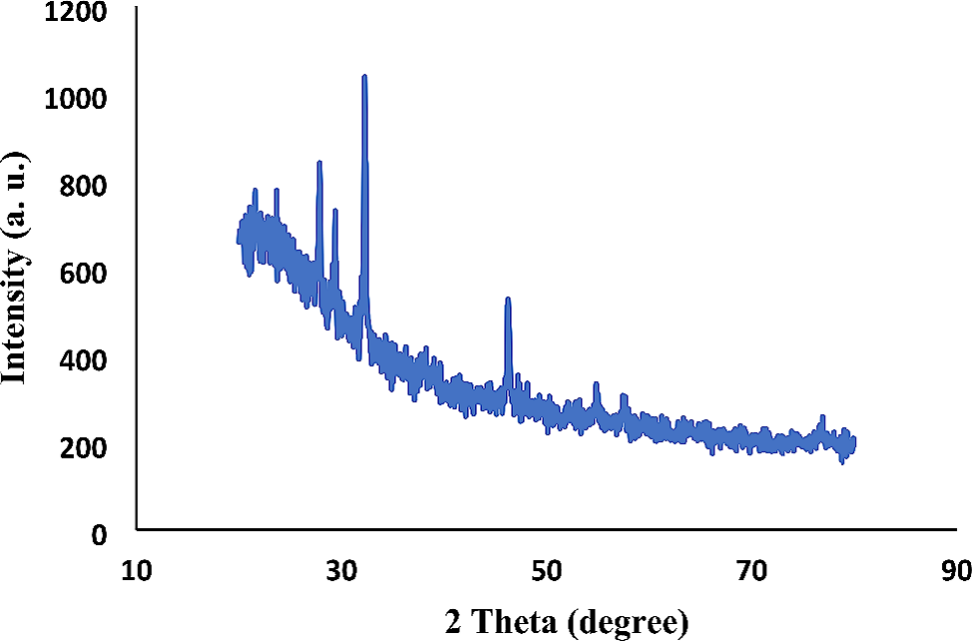
XRD pattern of synthesized nBG@Ag

**Fig. 2:**
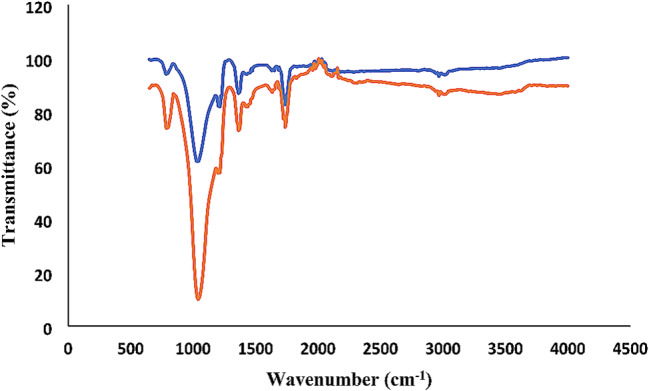
ATR-FTIR Spectra of nBG (Blue) and nBG@Ag (Red)

**Fig. 3:**
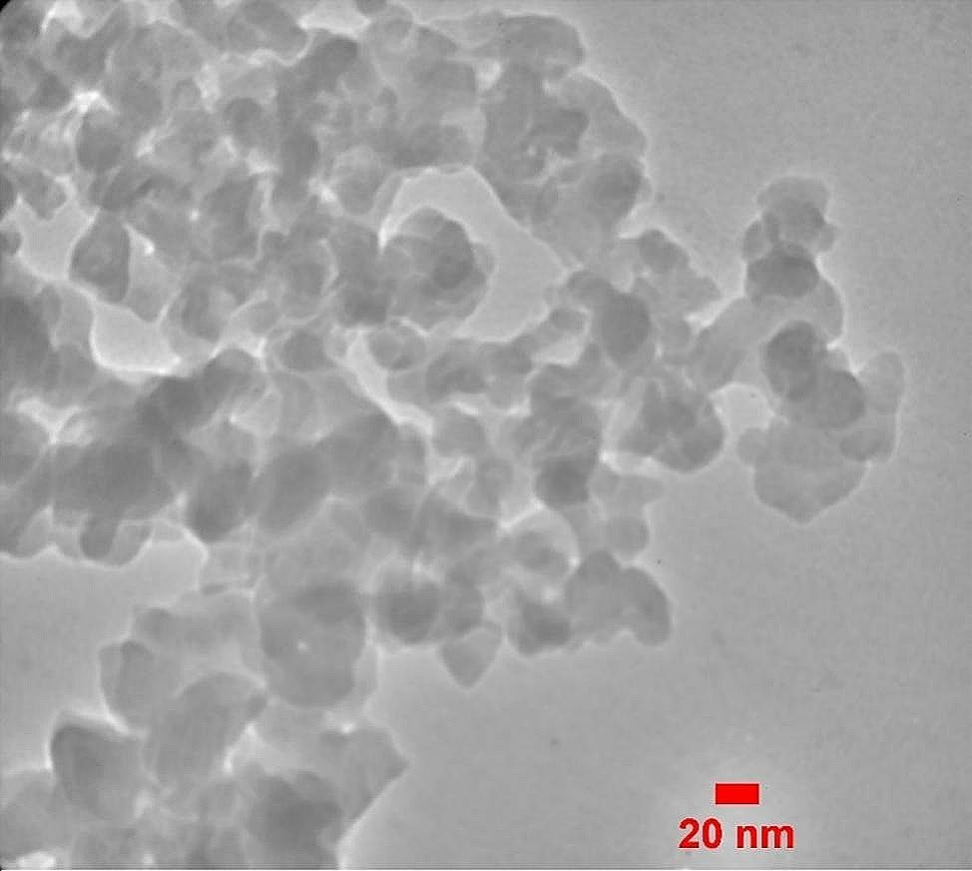
TEM image of synthesized nBG@Ag

The XRD peaks of the calcined nanoparticles at 650 ℃ correspond to the crystalline state of Larnite (Ca_2_SiO_4_) according to JCPDS # 33–0302. The peak observed at 2θ = 31.28 is in accordance with the Miller index (300), indicating significant crystallization of the synthesized nanoparticles in this region (Fig. 1) [11, 23]. The ATR-FTIR spectrum presented in Fig. 2 shows a broad band at 3410 cm^− 1^, indicating the presence of surface hydroxyl groups. The absorption bands at 1041 and 1036.4 cm^− 1^ correspond to the stretching and bending vibrations of the Si-O-Si group. Peaks at 791, 804, 792, and 812 cm^− 1^ correspond to the bending and stretching vibrations of the P-O group. Additionally, the absorption peak at 1616 cm^− 1^ belongs to the carbonate functional group. TEM image in Fig. 3 shows the relative aggregation of the synthesized bioactive glass. The TEM image also reveals that the size of the synthesized nanoparticles is less than 100 nanometers.

### Cytotoxicity

**Fig. 4:**
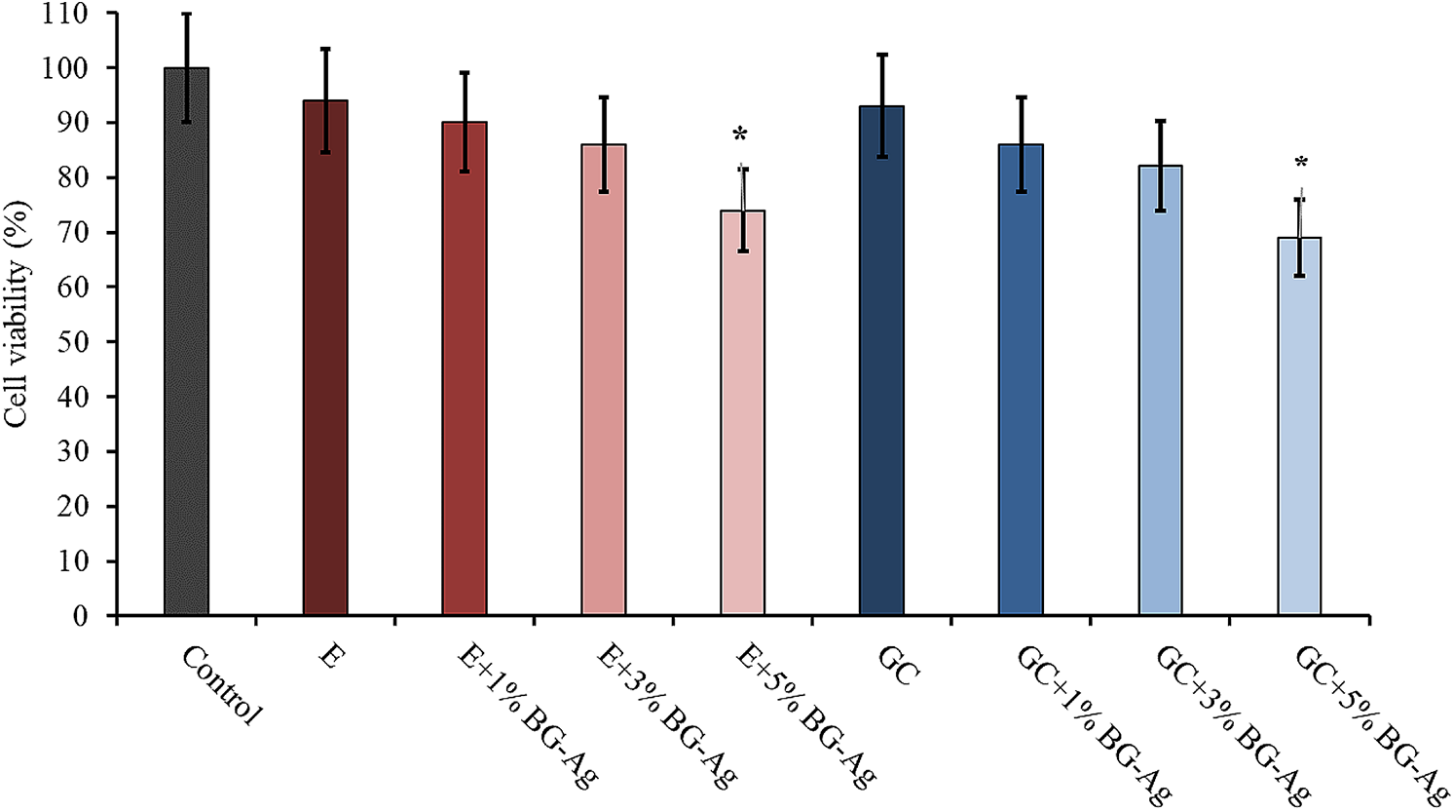
Cell viability of composites

The results of the biocompatibility study on the Vero cell line showed survival rates of 100, 94, 90, 86, 74, 93, 86, 82, and 69 for the control group (culture medium), groups 1, 2, 3, 4, 5, 6, 7, and 8, respectively. Although the survival rates of the samples treated with nBG@Ag decreased, this decrease was not statistically significant (Fig. 4).

### Antimicrobial activity

**Fig. 5:**
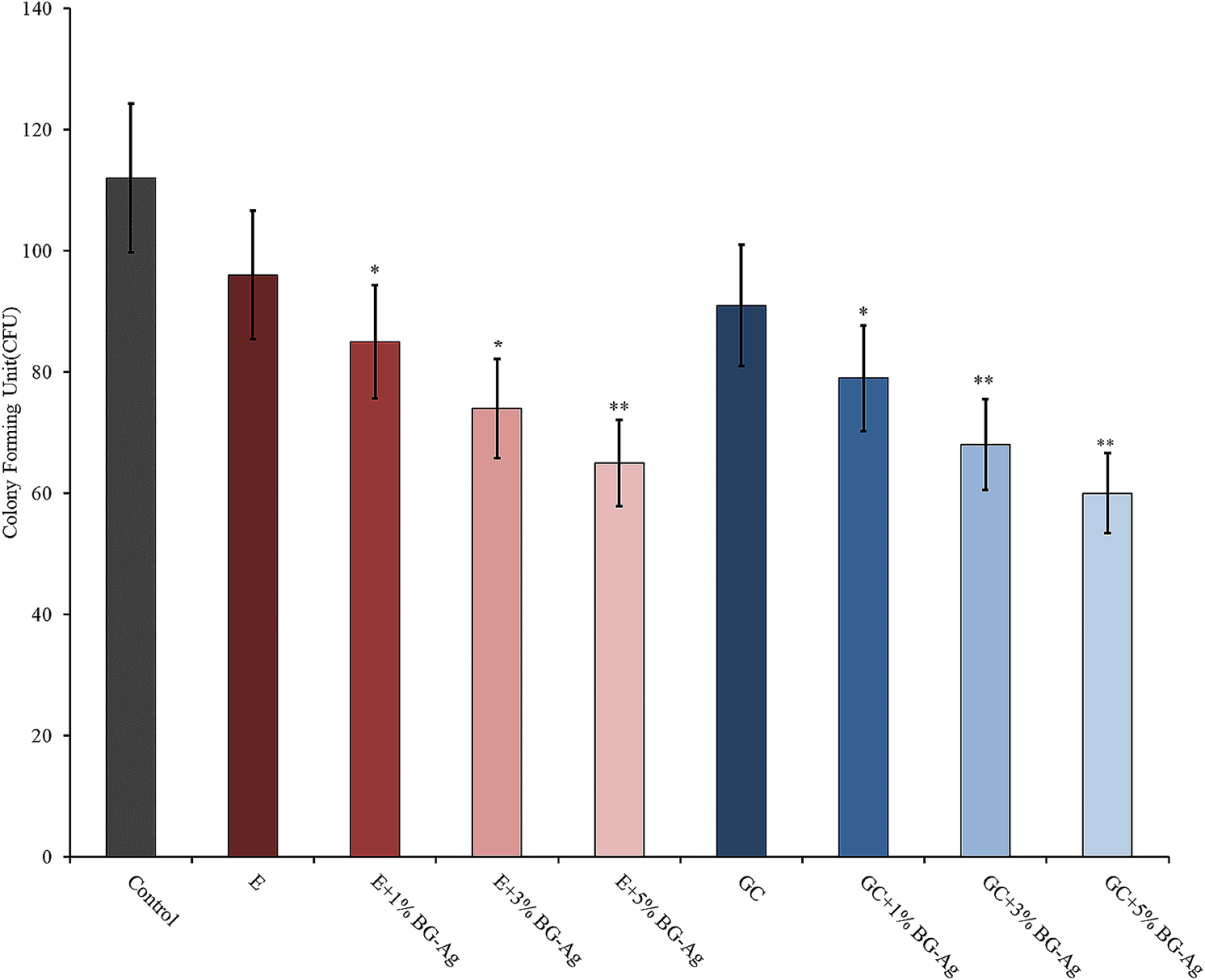
Antibacterial activity of composites (CFU/mL)

According to the results of the microbroth dilution method, the nBG@Ag solution exhibited the highest MIC of 0.125 µg mL^− 1^ and MBC of 0.25 µg mL^− 1^, whereas the BG solution showed zero µg mL^− 1^ for both cases. These findings indicate the inhibitory and bactericidal properties of the nBG@Ag compound. In the disk diffusion method, groups 2, 3, 4, 6, 7, and 8 showed a significant reduction in CFU/mL compared to the control group (*p* < 0.05). The 4th and 8th groups exhibited the highest reduction in CFU/mL. However, the reduction in CFU/mL was not statistically significant for the 1st and 5th groups compared to the control group (*p* > 0.05) (Fig. 5).

### Shear bond strength

**Fig. 6:**
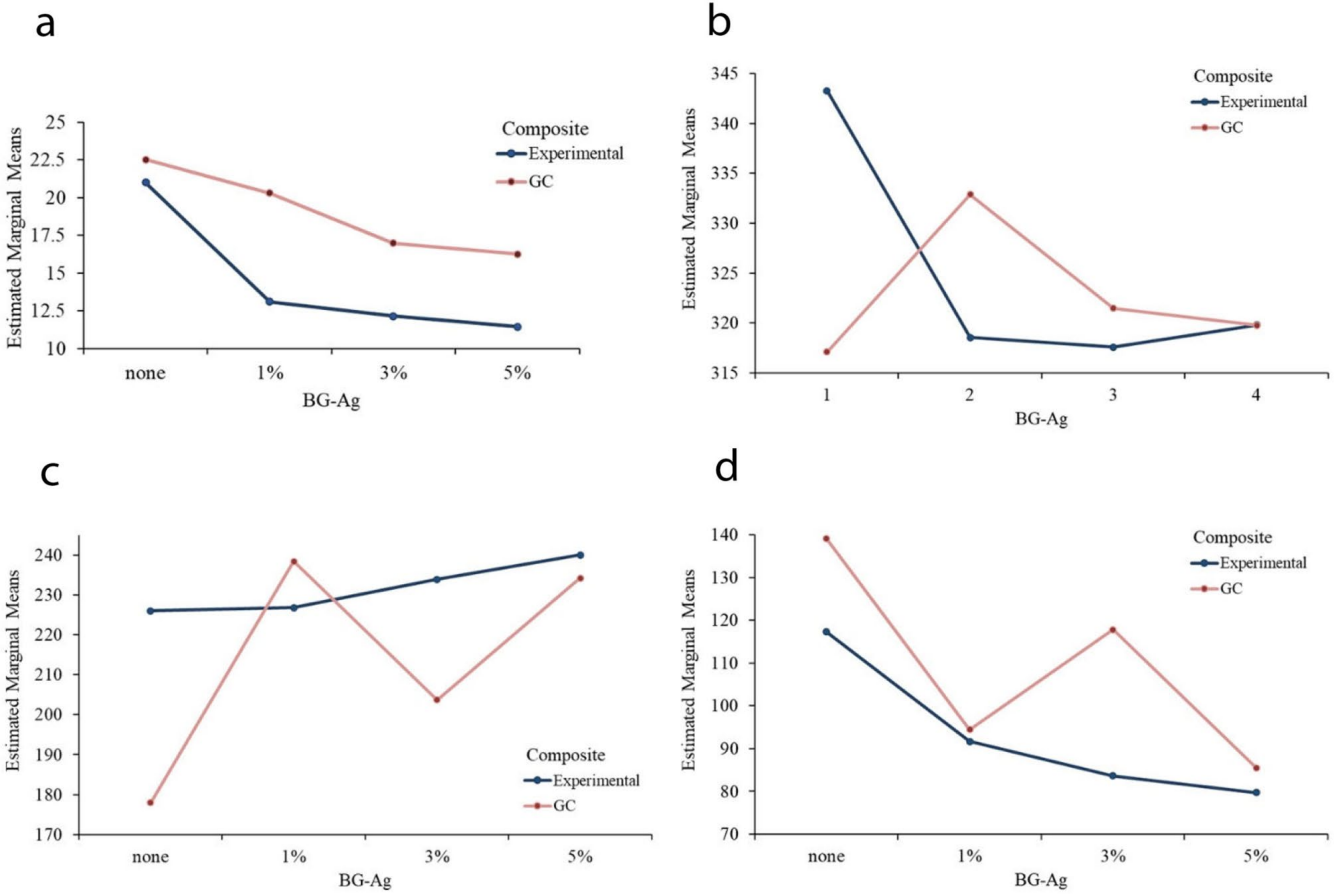
(**a**) Mean shear bond strength, (**b**) Initial microhardness of samples, (**c**) Secondary microhardness of samples, and (**d**) Changes in microhardness of samples in two types of composites based on different concentrations of nBG@Ag.

The statistical analysis of SBS values in the 8 examined groups is presented in Fig. 6a. Based on the Shapiro-Wilk test indicating the normal distribution of the data (*p* > 0.05). Since the equality of variances was accepted using the Levene’s test (*p* = 0.083), the mean SBS of the 8 groups was compared using one-way ANOVA. The results showed a statistically significant difference in the mean SBS among the 8 groups (*p* < 0.001). A pairwise comparison of SBS between groups using the Tukey-HSD method showed that group 1 had significantly higher SBS than groups 2, 3, and 4, but no significant difference was observed compared to groups 5, 6, 7, and 8. The SBS values in groups 2, 3, and 4 were significantly lower than groups 5 and 6. In group 5, the SBS value did not show a statistically significant difference compared to groups 6 and 7, but it was significantly higher than group 8. Additionally, there was no statistically significant difference in SBS between groups 6 and 7, as well as between groups 7 and 8. Two-way ANOVA was used to investigate the effect of composite and bioactive glass-silver on SBS. The results showed a statistically significant effect of composite on SBS (*p* < 0.001), with GC composite having higher SBS values compared to Experimental composite. The effect of bioactive glass-silver on SBS was also significant (*p* < 0.001). Pairwise comparison of bioactive glass-silver using the Tukey-HSD method showed that while the different percentages of bioactive glass-silver (1%, 3%, and 5%) did not have a statistically significant difference, all three bioactive glass-silver groups had lower SBS values compared to the group without bioactive glass-silver (control). The control group compared to the group containing 1% bio-glass-silver has a p-value of 0.002, and compared to the groups containing 3% and 5% bio-glass-silver, it has a p-value of less than 0.001. The comparison between the groups containing different percentages of bio-glass-silver (1%, 3%, and 5%) is not significant.

Furthermore, there is no significant interaction effect between the composite and bio-glass-silver (*p* = 0.214). As shown in Fig. 6a, the reduction in SBS behavior at different percentages of bio-glass-silver in two types of composites is nearly similar.

### Surface microhardness

The statistical analysis of initial microhardness in the 8 examined groups in Fig. 6b are presented. According to the Shapiro-Wilk test, confirming the normal distribution of data in each of the 8 groups (*p* > 0,05). Therefore, the comparison of microhardness among the 8 groups before intervention is performed using one-way analysis of variance (ANOVA). The equality of variances of the groups was checked using the Levene test, and it was confirmed (*p* = 0.153) that the variances were homogeneous. The one-way ANOVA results showed that the 8 examined groups before intervention did not have statistically significant differences (*p* = 0.753). The statistical analysis of secondary microhardness variables, separated by the examined groups, are presented in Fig. 6c. According to the Shapiro-Wilk test, confirming the normal distribution of data in each of the 8 groups (*p* > 0,05). According to the result of the Levene test, the equality of variances among the groups was not confirmed with a p-value of 0.020. Therefore, the comparison of means in the 8 groups based on secondary microhardness was performed using the Robust-Welch test. The results showed that secondary microhardness does not have statistically significant differences in the 8 groups (*p* = 0.540). Despite the lack of statistically significant differences in initial microhardness among the groups, since in some groups, microhardness differed by about 35 units compared to other groups, to adjust the differences and make a more accurate comparison of microhardness among the groups, the amount of microhardness changes was calculated, and the statistical analysis of the differences in microhardness among the groups are presented in Fig. 6d.

Since, according to the Levene test, the equality of variances of the changes in microhardness in the 8 groups was confirmed (*p* = 0.380), the comparison of the means of changes in microhardness in the groups was examined using one-way ANOVA, and the results showed that the changes in microhardness in the 8 groups do not have statistically significant differences (*p* = 0.712). Within-group comparisons of microhardness were conducted using paired t-tests. A statistically significant decrease in microhardness was observed in all groups, respectively. To investigate the effect of each of the composite and bio-glass-silver variables, a two-way ANOVA was used. The results showed that neither on secondary microhardness nor on the changes in tooth microhardness, composite and nBG@Ag had statistically significant effects. Furthermore, there was no significant interaction effect between composite and bio-glass-silver.

### Examination of sample remineralization using SEM

**Fig. 7:**
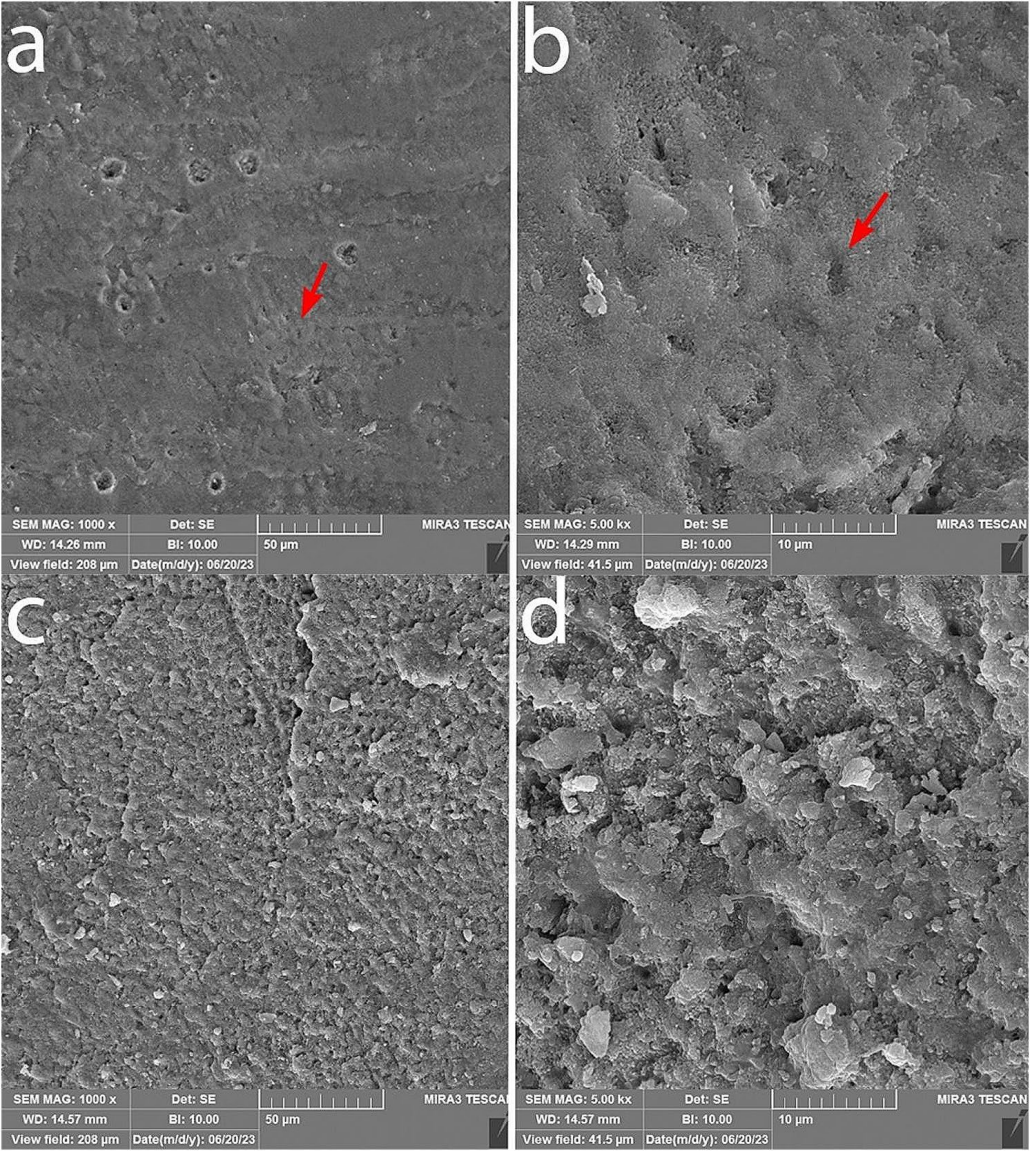
SEM images of the enamel surface of teeth bonded with experimental adhesives: (**a**) Adhesive without nBG@Ag at ×1000 magnification. (**b**) The same sample as (**a**) at ×5000 magnification. (**c**) Adhesive containing 5% nBG@Ag at ×1000 magnification. (**d**) The same sample as (**c**) at ×5000 magnification

**Fig. 8:**
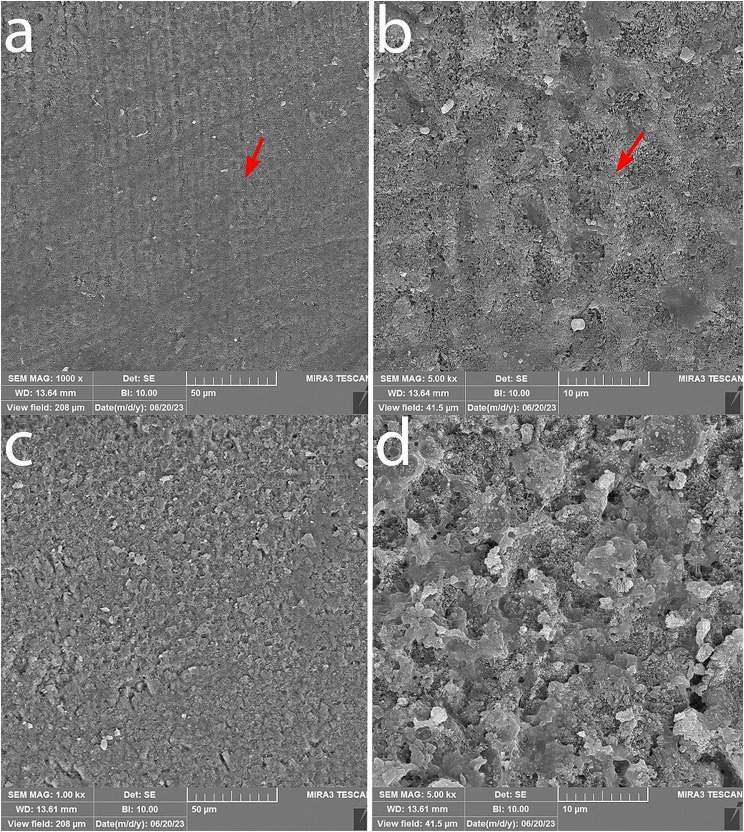
SEM images of the enamel surface of teeth bonded with GC adhesives: (**a**) Adhesive without nBG@Ag at ×1000 magnification. (**b**) The same sample as (**a**) at ×5000 magnification. (**c**) Adhesive containing 5% nBG@Ag at ×1000 magnification. (**d**) The same sample as (**c**) at ×5000 magnification

**Fig. 9:**
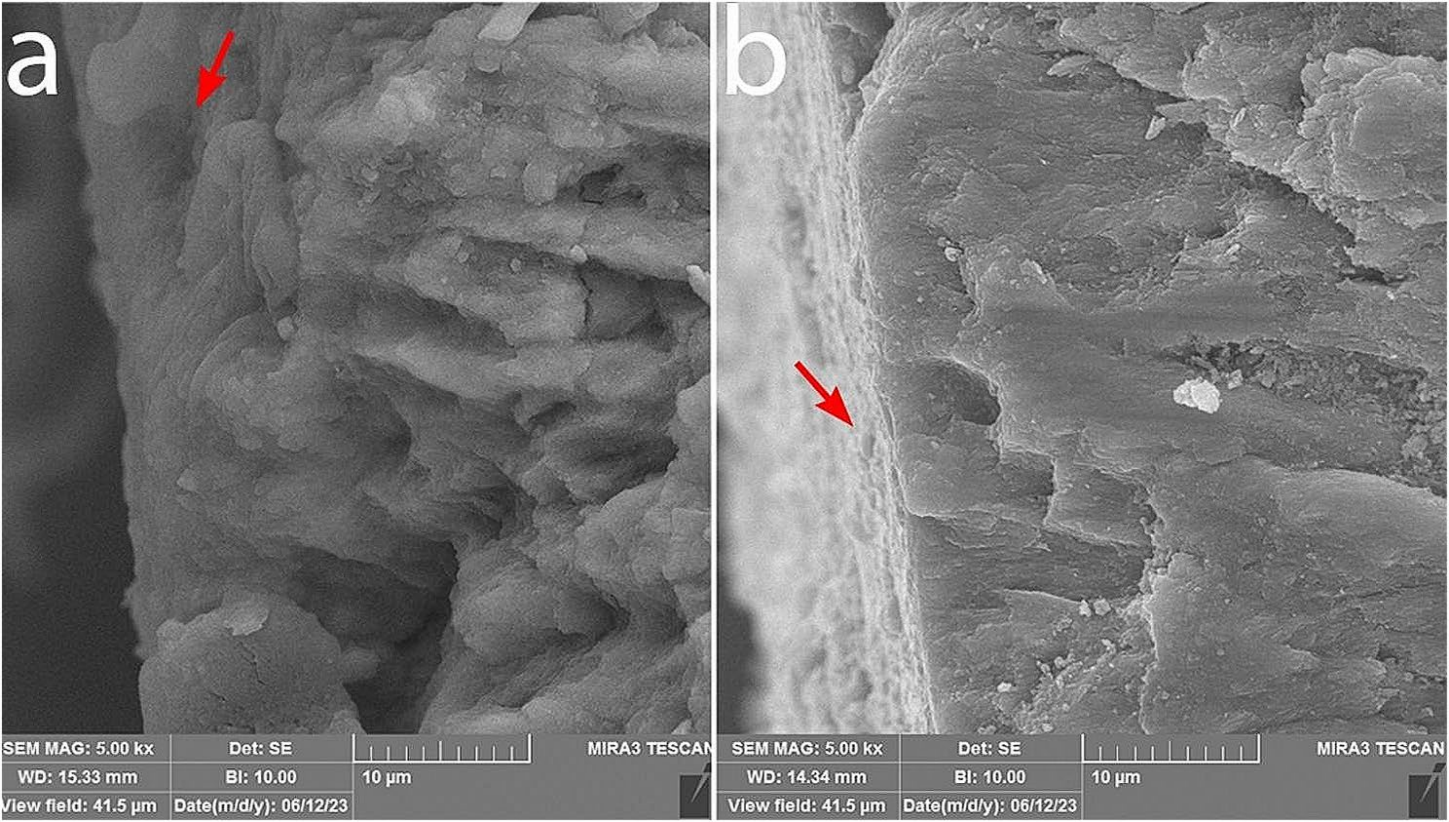
SEM cross-sectional images of samples bonded with GC adhesives at ×5000 magnification: (**a**) Adhesive without nBG@Ag. (**b**) Adhesive containing 5% nBG@Ag.

**Fig. 10:**
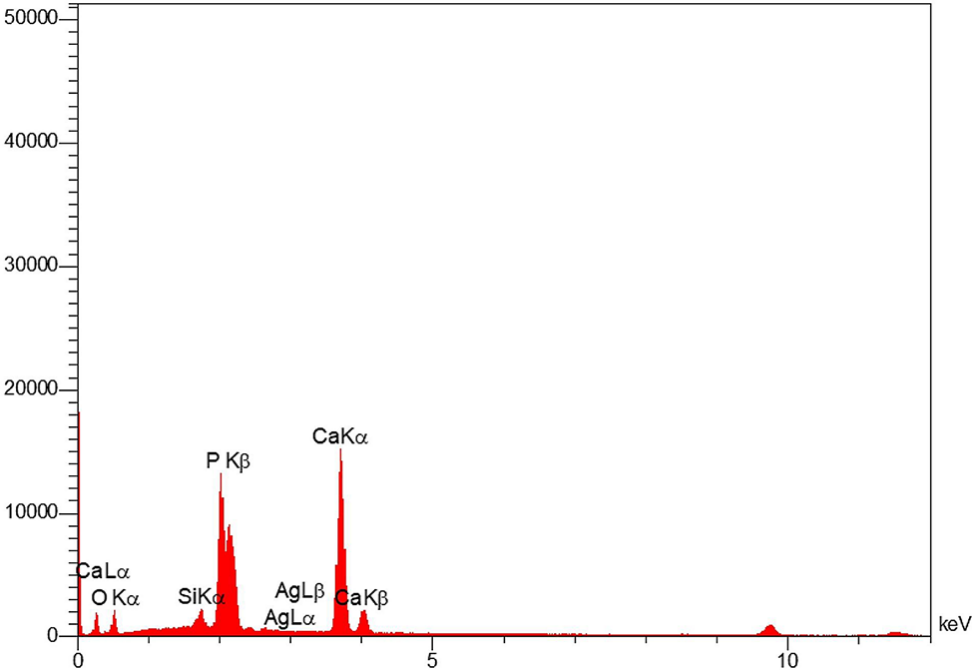
EDX of the sample bonded with GC adhesive containing 5% nBG@Ag.

The surface morphology was examined using a SEM microscope with ×1000 and ×5000 magnification in Figs. 7 and 8. Honeycomb-like appearance is visible on the surface of samples bonded with the control composites, resulting from surface enamel demineralization. In the samples bonded with experimental composite containing 5% nBG@Ag and GC composite containing 5% nBG@Ag, porous enamel structures are covered with a layer of hydroxyapatite crystal deposits. In cross-sectional images at ×5000 magnification of samples bonded with GC control composite, porous enamel structures are visible, and in the image of the sample bonded with GC composite containing 5% nBG@Ag, surface coverage with a layer of hydroxyapatite deposits is observable (Fig. 9). Additionally, in the elemental analysis spectrum (EDS) of the sample, it can be observed that this layer is primarily composed of calcium and phosphorus, with trace amounts of silica and silver (Fig. 10).

## Discussion

This study aimed to investigate the potential of preventing white spot lesions by incorporating nano-bioactive glass (nBG) doped with silver particles into orthodontic adhesives. The antimicrobial properties and remineralization efficacy of these adhesives were evaluated. Additionally, the clinical applicability of these adhesives was assessed through mechanical property testing and toxicity evaluation. The results of this study showed that the adhesives containing nBG@Ag were highly biocompatible, as there were no significant differences in cytotoxicity compared to the control group. The inclusion of nBG@Ag in the composite adhesives led to a significant reduction in the *S. mutans* growth, as a bacteria associated with early carious lesions. Moreover, the antibacterial activity increased with higher percentages of nBG@Ag in the adhesive. Although the shear bond strength of the adhesives decreased significantly after the addition of nBG@Ag, it remained above the recommended value for bracket bonding. Furthermore, the addition of nBG@Ag showed improvement in the microhardness of the teeth.

Characterization analysis confirmed the crystalline structure, chemical composition, and size of the synthesized nanomaterials. The orthodontic adhesives incorporating nBG@Ag demonstrated no cytotoxicity to cellular structures, while exhibiting enhanced antibacterial activity compared to controls. Moreover, the shear bond strength assessment indicated that the addition of nBG@Ag to two different types of orthodontic adhesives yielded satisfactory values, suggesting their potential as bonding agents in clinical settings. Furthermore, the study revealed a modest enhancement in enamel lesion remineralization when using adhesives containing nBG@Ag. Surface characterization via SEM illustrated improvements in sample surface characteristics, further corroborating the potential clinical utility of these novel adhesives.

The findings of the present investigation concerning cytotoxicitym, as determined via the MTT assay, indicated that the incorporation of 5% *w/w* of nano-bioactive glass containing silver into the composite resulted in Vero cell survival rates in the nBG@Ag groups comparable to those observed in the control group. While a slight decrease in cell survival was observed with increasing percentages of nBG@Ag, this decrement did not attain statistical significance, underscoring the exceptionally high biocompatibility of the synthesized nano-structured bioactive glass.

Lee et al. [18] conducted a study exploring the remineralization properties of an orthodontic primer containing silver-enhanced bioactive glass. Consistent with the present study, they observed comparable cell survival rates between the control and study groups at 24 h, albeit noting a slight decline in cell survival at 48 h. In a study by Jung et al. [32], the antibacterial effects and dentin sealing properties of nano-composites containing 1%, 3%, and 5% silver-enhanced bioactive glass coated with mesoporous silica (Ag-BGN@MSN) were investigated. The combination utilized in their study demonstrated a relative cell survival rate exceeding 72%, aligning with the outcomes of the current investigation. Furthermore, Song et al. [20] assessed the cellular toxicity of orthodontic resins containing nano-bioactive glass doped with gallium, utilizing dental pulp stem cells. Their findings in in agreement with those of the present study, further reinforcing the biocompatibility of such formulations.

In the assessment of antibacterial activity, the inclusion of nBG@Ag exhibited notable inhibition of *S. mutans* growth in comparison to the control group, indicating its potential utility in caries prevention. Several factors contribute to this effect, including the alkaline property recognized as the primary antibacterial mechanism of bioactive glass. Moreover, smaller particle sizes can augment surface area, potentially intensifying the antibacterial effect. Additionally, the presence of silver ions, renowned for their antimicrobial properties, likely contributes significantly to the observed antibacterial effects. Discs fabricated from composites containing bioactive glass-silver in both Experimental and GC compositions demonstrated a significant reduction in *S. mutans* growth. Furthermore, the antibacterial activity escalated with an increase in the percentage of bioactive glass-silver. However, the observed elevation in antibacterial efficacy was not statistically significant across composites containing varying proportions of nBG@Ag (1%, 3%, and 5%). Kim et al. [15] conducted a study investigating the antibacterial activity of orthodontic bonding materials containing bioactive glass against *S. mutans*, aligning with the findings of this research. Similarly, Choi et al. [14] examined self-etching orthodontic resin incorporating bioactive glass, revealing a significant enhancement in composite antibacterial efficacy against *S. mutans* and *P. gingivalis*, with no notable differences observed among varying percentages of added bioactive glass. Furthermore, Jung et al. [32] corroborated these observations by demonstrating the antimicrobial activity of silver-doped bioactive glass against *Lactobacillus casei* in their study. These findings underscore the promising antimicrobial potential of bioactive glass-based formulations in orthodontic applications.

In ideal circumstances, the shear bond strength (SBS) of metal brackets to tooth enamel should fall within the range of 7–9 megapascals (MPa), ensuring adequate bonding while facilitating bracket removal post-orthodontic treatment [27]. In our study, the SBS values across all groups surpassed this threshold, thus meeting clinical acceptability criteria. Upon comparison between the GC composite and the Experimental-synthesized composite, it was noted that SBS values were significantly higher in all corresponding groups of the GC composite. In both composite types utilized, the addition of bioactive glass-silver resulted in a decline in SBS, with this reduction becoming more pronounced with escalating percentages of bioactive glass-silver. Notably, this decrease was more conspicuous in the Experimental-synthesized composite. This decline in SBS may be attributed to the presence of bioactive glass-silver nanoparticles within the polymer matrix, potentially disrupting the polymerization process and consequently diminishing the degree of polymerization and mechanical properties. Moreover, nanoparticle agglomeration within the composite could act as stress concentration points, further compromising shear bond strength [33]. This reduction in SBS aligns with the findings of a systematic review by Poorhajibagher et al. [34].

Choi et al. [14] conducted a study, utilizing a self-etching orthodontic resin devoid of a primer to examine the antibacterial and remineralizing properties of orthodontic composites containing varying percentages (1%, 3%, and 5% *wt/wt*) of porous bioactive glass (MBN). They observed an increase in shear bond strength for samples containing 1% and 3% MBN, but a decrease for those containing 5% MBN. Similarly, our study demonstrated the detrimental impact of 5% nBG@Ag on shear bond strength; however, unlike the aforementioned study, a reduction in SBS was observed in samples containing 1% and 3% bioactive glass-silver. This disparity in results may stem from differences in the composition of the additives used in the composites. Lee et al. [18] reported an increase in shear bond strength in primer groups containing silver and zinc-doped bioactive glass, further substantiating the influence of composition on SBS. Additionally, Kohda et al. [16] investigated the enamel demineralization prevention and shear bond strength properties of a resin incorporating varying proportions of bioactive glass, noting a decrease in SBS with increasing bioactive glass content, akin to our findings. Furthermore, Eslamian et al. [35] observed a significant reduction in SBS with the addition of 0.3% silver nanoparticles to the adhesive in their study.

The examination of secondary microhardness in teeth post pH cycling, juxtaposed with initial microhardness, revealed a significant reduction across all study groups. However, the variations in microhardness between the study groups did not reach statistical significance. Despite these observations, the results indicate that the addition of bioactive glass-silver to the adhesive led to a lesser reduction in microhardness compared to the control group in the samples. Furthermore, in the synthesized adhesive compared to the GC adhesive, a less significant decrease in tooth microhardness was observed with an increase in the percentage of nBG@Ag. Manfred et al. [36] similarly demonstrated enhanced demineralization prevention with the addition of bioactive glass. Rahmanipanah et al. [37] evaluated shear bond strength and enamel remineralization using an orthodontic composite containing nano-hydroxyapatite, revealing that exposure to a remineralizing solution resulted in a lesser reduction in microhardness in groups with added nanoparticles, consistent with our findings. Kim et al. [15] showed the preventive effects of orthodontic adhesive containing bioactive glass on enamel demineralization within 200–300 micrometers from the bracket’s margin. Lee et al. [18] showed that addition of 0.2% of BG-Ag and BG-Zn to the adhesive did not yield a significant difference compared to the control group. However, an increase in the percentage of BG-Ag and BG-Zn to 1% demonstrated a significant difference in remineralization, with the highest remineralization observed in the adhesive containing 1% BG-Ag. Similarly, Choi et al. [14] reported a significant increase in remineralization with the addition of bioactive glass to self-adhesive orthodontic resin, corroborating findings by Song et al. [20] on orthodontic resins containing gallium-doped bioactive glass. In our study, superior remineralization outcomes were observed in composites containing bioactive glass-silver compared to the control group. Disparate findings post pH cycling may be attributed to methodological variances in measuring remineralization.

In this study, SEM was employed to assess the quality of enamel remineralization. In samples bonded with both GC adhesives and Experimental adhesives containing 5% nBG@Ag, hydroxyapatite crystals uniformly covered the enamel surfaces, rendering the honeycomb-like appearance typically associated with demineralized enamel surfaces invisible. Conversely, in samples bonded with GC adhesives and Experimental adhesives devoid of bioactive glass-silver (control groups), the honeycomb-like appearance on enamel surfaces was distinctly observable. Thus, SEM evaluation corroborated the findings of the microhardness test, indicating an enhancement in remineralization. Narayana et al. [38] explored the effect of bioactive glass remineralization on artificially induced caries lesions, with SEM images in their study exhibiting a uniform distribution of mineral deposits on the enamel surface, akin to the observations in our study. Similarly, Güçlü et al. [39] employed SEM to confirm the remineralization of white spot lesion surfaces using bioactive glass. However, in contrast to our findings, their study also noted areas of remaining exposed enamel on the enamel surfaces. These variations in outcomes may stem from differences in experimental protocols, sample preparation techniques, or the specific formulations of bioactive glass utilized.

A notable limitation of this study is the absence of separate groups subjected to thermocycling to evaluate bond strength. This limitation arose due to the inclusion of eight experimental groups, resulting in a substantial increase in the number of required samples. As this study was conducted under controlled laboratory conditions, it is recommended to conduct a clinical study to validate the findings. Clinical studies are imperative as they encompass a broader array of influencing factors not accounted for in laboratory settings. These factors include temperature and pH variations in oral saliva, the impact of diverse bacterial species beyond *S. mutans*, dietary habits, and oral hygiene practices.

## Conclusion

In conclusion, the application of nanostructured nBG@Ag in primerless orthodontic adhesives (self-adhesive) offers a promising approach. It demonstrates high biocompatibility while maintaining mechanical properties, particularly shear bond strength. This innovative composition promotes the formation of a hydroxyapatite layer on enamel surfaces, resulting in enhanced antimicrobial properties and reduced enamel demineralization. Consequently, it holds the potential to significantly decrease the occurrence of white spot lesions in the vicinity of orthodontic brackets. Further clinical investigations are warranted to validate these findings and assess their real-world applicability.

## Data Availability

Data supporting this study are included within the article.
